# Bone tissue engineering using adipose‐derived stem cells and endothelial cells: Effects of the cell ratio

**DOI:** 10.1111/jcmm.15374

**Published:** 2020-05-12

**Authors:** Hilkea Mutschall, Sophie Winkler, Volker Weisbach, Andreas Arkudas, Raymund E. Horch, Dominik Steiner

**Affiliations:** ^1^ Department of Plastic and Hand Surgery University Hospital of Erlangen Friedrich‐Alexander‐University Erlangen‐Nürnberg (FAU) Erlangen Germany; ^2^ Department of Transfusion Medicine University Hospital of Erlangen Friedrich‐Alexander‐University Erlangen‐Nürnberg (FAU) Erlangen Germany

**Keywords:** ADSC, angiogenesis, bone tissue engineering, HUVEC, osteogenesis

## Abstract

The microvascular endothelial network is essential for bone formation and regeneration. In this context, endothelial cells not only support vascularization but also influence bone physiology via cell contact‐dependent mechanisms. In order to improve vascularization and osteogenesis in tissue engineering applications, several strategies have been developed. One promising approach is the coapplication of endothelial and adipose derived stem cells (ADSCs). In this study, we aimed at investigating the best ratio of human umbilical vein endothelial cells (HUVECs) and osteogenic differentiated ADSCs with regard to proliferation, apoptosis, osteogenesis and angiogenesis. For this purpose, cocultures of ADSCs and HUVECs with ratios of 25%:75%, 50%:50% and 75%:25% were performed. We were able to prove that cocultivation supports proliferation whereas apoptosis was unidirectional decreased in cocultured HUVECs mediated by a p‐BAD‐dependent mechanism. Moreover, coculturing ADSCs and HUVECs stimulated matrix mineralization and the activity of alkaline phosphatase (ALP). Increased gene expression of the proangiogenic markers eNOS, Flt, Ang2 and MMP3 as well as sprouting phenomena in matrigel assays proved the angiogenic potential of the coculture. In summary, coculturing ADSCs and HUVECs stimulates proliferation, cell survival, osteogenesis and angiogenesis particularly in the 50%:50% coculture.

## 1 INTRODUCTION

The reconstruction of critical sized bone defects can be challenging in clinical practice. Critical bone defects can be caused by malformation, cancer, trauma or infection. Regardless of the entity, the current gold standard is autologous tissue transfer, which can be associated with significant donor side morbidity and limited tissue availability. One way to circumvent these problems is the generation of bioartificial bone tissue.

For bone formation and regeneration, a sufficient vascularization providing oxygen and nutrition supply is indispensable.^1^, ^2^, ^3^ Strategies to improve vascularization in bone tissue engineering applications include the use of angiogenic growth factors, endothelial cells (ECs) and the surgical induced angiogenesis by means of arteriovenous loops.^4^, ^5^, ^6^ In many studies, the cocultivation of ECs and mesenchymal stem cells (MSCs) has already proven to be beneficial for proliferation and osteogenic differentiation.^7^, ^8^, ^9^, ^10^, ^11^ With regard to clinical practice, the isolation of MSCs from the bone marrow can be limited in terms of quantity and donor side morbidity. Stem cells from fat tissue are an interesting alternative to MSCs derived from bone marrow. Isolation, characterization and multiple differentiation potential have already been described in the literature, and it has been shown that adipose‐derived stem cells (ADSCs) are also suitable for bone defect healing in animals.^12^, ^13^ In addition to that, it has been shown that ADSCs and bone marrow MSCs have both osteogenic differentiation potential.^14^, ^15^, ^16^ Furthermore, ADSCs are even superior to MSCs in terms of immunomodulatory capabilities and secretion of proangiogenic factors and extracellular matrix components.^17^, ^18^, ^19^, ^20^, ^21^

Especially in coculture with endothelial cells, the osteogenic differentiation of ADSCs can be further increased.^22^

Moreover, human umbilical vein endothelial cells (HUVECs) have a pronounced vascularization capacity.^5^, ^21^, ^23^, ^24^ The coapplication of ADSCs and HUVECs in terms of tissue engineering applications seems to be a promising approach to increase vascularization and bone formation.

The aim of this study was to investigate the optimal ratio of HUVECs and osteogenic differentiated ADSCs in the two‐dimensional cell culture and the effects on proliferation, cell survival, osteogenesis and angiogenesis. Using negative immunoselection, we tried to enlighten the cell type specific effects regarding apoptosis, angiogenesis and osteogenic differentiation more in detail.

## 2 MATERIALS AND METHODS

### 2.1 Cell culture

Human ADSCs were isolated from five patients undergoing autologous breast reconstruction, according to an established protocol.^25^ The biological material was received with the informed consent of the patients, according to hospital's ethics committee guidelines [AZ: 126_16]. ADSCs were cultured in MEMa (Gibco), supplemented with 10% FCS superior (Biochrom), 100 U/mL penicillin and 100 µg/mL streptomycin (Biochrom). The isolated ADSCs were positive for CD105 (99.68 ± 0.13%) as well as CD90 and CD73 (99.92 ± 0.04%) in a subsequent FACS analysis.

Human umbilical vein endothelial cells (PromoCell) were cultured in Endothelial Cell Growth Medium (ECGM) (PromoCell), supplemented with 10% FCS superior, 100 U/ml penicillin, 100 µg/mL streptomycin and supplements. Both cells types were cultured under humidified conditions (37°C, 5% CO_2_). Medium was changed two times a week and all cells were used until passage 5.

ADSCs underwent osteogenic differentiation for 14 days, before coculturing with HUVECs. To induce osteogenic differentiation, ADSCs were cultured in ECGM medium, modified with 50 µg/mL L‐ascorbic acid, 10 mmol/L glycerophosphate, 1 × 10^−8^ mol/L dexamethasone, 0.01 µmol/L 1,25‐dihydroxyvitamine D3 (all supplements purchased from Sigma), 10% FCS superior, 100 U/mL penicillin and 100 µg/mL streptomycin, according to an established protocol.^26^

To determine the ideal concentration of ADSCs and HUVECs, five groups were formed, the monoculture of ADSCs and HUVECs and cocultures with different ratios (75%:25%, 50%:50% and 25%:75%). All experiments were performed with the ADSCs from the 5 donors in a concentration of 5000 cells/cm^2^ with osteogenic modified ECGM. The cells were grown on plastic cell culture plates as two‐dimensional cell cultures.

### 2.2 Cell viability assay

After 3, 7 and 14 days, cell viability was assessed using a WST‐8 assay. After refreshing medium and adding CCVK‐I‐Solution (PromoCell), all five groups were incubated for 2 hours at 37°C. The resulting supernatant was analyzed photometrically by using an ELISA Reader at 450 nm.

### 2.3 Negative immunoselection

**FIGURE 1:**
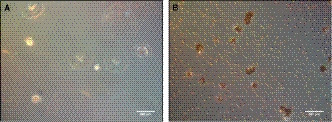
Detached ADSCs and HUVECs after negative immunoseparation. Separated ADSCs (A) and HUVECs (B). The latter ones were coated with CD31 magnetic beads

In order to describe celltype specific effects, negative immunoselection was carried out after 3, 7 and 14 days. Briefly, cells were detached from cell culture dishes, resuspended in 1 mL PBS containing 0.1% BSA and 25 µL magnetic beads, coated with CD31 antibody (Invitrogen). After incubation for 20 minutes at 4°C, cells were separated, using a magnetic separator (DynaMg™, Invitrogen). The unbound ADSCs were transferred into an Eppendorf Cup, while the HUVECs remained in the magnetic separator (Figure 1). Finally, the cells were used for further experiments (see 2.4, 2.5 and 2.10).

### 2.4 Cell death detection Assay

To quantify the DNA fragmentation in apoptotic cells, a cell death detection ELISA (Sigma) was performed after 7 and 14 days. As recommended by the manufacturer, cells were resuspended in 200 µL lysis buffer after negative immunoselection. Subsequently, samples were centrifuged at 200 x *g* for 10 minutes. 20 µL of the resulting supernatant were transferred into an anti‐histone antibody‐coated microplate. After adding the peroxidase‐labelled anti‐DNA antibody and the ABTS substrate (2,2′‐Azino‐di[3‐ethylbenzthiazolin‐sulfonat]), the absorbance was measured at 405 nm.

**TABLE 1:** The primer sequences used for polymerase chain reaction (PCR)

Gene	Primer sequence (5′‐3′)
MMP3‐F	GCCATCTCTTCCTTCAGGCG
MMP3‐R	TCACGGTTGGAGGGAAACCT
Ang2‐F	GGGCATAATTGTGCTTGACTGG
Ang2‐R	GCCGTTCGAACTGTCTCACC
Flt1‐F	AGGATTACCCGGGGAAGTGG
Flt1‐R	AGAGTCCGTCCTCTCGTTCG
eNOS‐F	CGAGTGAAGGCGACAATCCT
eNOS‐R	CGAGGGACACCACGTCATAC
RPL13a‐F	CCTTCCTCCATTGTTGCCCT
RPL13a‐R	TGCACAATTCTCCGAGTGCT
ALP‐F	CCAAGGACGCTGGGAAATCT
ALP‐R	TATGCATGAGCTGGTAGGCG
RUNX2‐F	AGAGTCCTTCTGTGGCATGC
RUNX2‐R	CTTGGGTGGGTGGAGGATTC

Abbreviations: ALP =alkaline phosphatase; Ang2= angiopoetin 2; eNOS= endothelial nitric oxidase; F= Forward Primer; Flt= VEGF receptor 1; MMP3= matrix metalloproteases 3; R= Reverse Primer; RPL13a= ribosomal protein L13; RUNX2= runt‐related transcription factor 2.

### 2.5 Quantification of phosphorylated BAD

The phosphorylation of the proapoptotic protein BAD was assessed by using a p‐BAD Sandwich ELISA (Cell Signaling Technology) after 7 days. According to manufacturer specifications, cells were resuspended in 300 µL ice‐cold lysis buffer, supplemented with PMFS, mechanical lysed and centrifuged at 18 800 x *g* for 10 minutes at 4°C. Afterwards, 100 µL of the resulting supernatant were applied to an ELISA plate and incubated overnight at 4°C. After a washing step, the detection antibody was added, and the microplate incubated for 1 hour at 37°C. Thereafter, the HRP‐linked antibody was applied, the plate incubated for 30 minutes at 37°C and after additional wash steps the TMB substrate added. The reaction was stopped by adding the stop solution and absorbance measured at 450 nm.

### 2.6 Alizarin red assay

Matrix mineralization was measured with an Alizarin Red‐based assay after 14 days, according to manufacturer recommendation. Briefly, samples were fixed with 4% paraformaldehyde. After fixation, the samples were washed with phosphate‐buffered saline (PBS) (Sigma). Then, 1 mL Alizarin Red staining solution (ScienCell) was added. After incubation for 30 minutes, the dye was removed, and the samples washed and 800 µL acetic acid (ScienCell) added. Afterwards, the cells were collected using a cell scraper and the samples heated at 85°C for 10 minutes. After cooling and centrifugation, the supernatant was collected, neutralized using 10% ammonium hydroxide (ScienCell), and absorbance measured using an ELISA Reader at 405 nm.

### 2.7 Quantification ALP activity

Osteogenic differentiation was assessed by alkaline phosphatase (ALP) activity. The ALP assay (Abcam) was performed after 3 and 7 days according to manufacturer information. Briefly, the cells were detached by using Accutase^®^ solution (Sigma), washed with ice‐cold PBS, centrifuged at 300 × *g* for 4 minutes, resuspended in 200 µL assay buffer and centrifuged again at 18 800 × *g* for 15 minutes. Afterwards, the resulting supernatant was transferred into microtitre plates and the *p*NPP solution added. After 60 minutes, a stop solution was added and absorbance measured at 405 nm.

### 2.8 Matrigel assay

To investigate the angiogenic potential of cocultures containing ADSCs and HUVECs, a matrigel assay was performed. 10 µL matrigel (Corning) were pipetted into each well of a µ‐Slice (ibidi). After polymerisation for 30 minutes at 37°C, 10 000 cells per well were added and incubated for 4 hours, at 37°C, 5% CO_2_. Vital cells were visualized using calcein staining (Sigma). The number of branches and the length of vessel network were analyzed by Angiogenesis Analyzer (ImageJ version 2 NIH).

### 2.9 VEGF ELISA

The amount of VEGF in the cell culture supernatant was investigated by a VEGF ELISA (R&D Systems) after 3 and 7 days according to manufacturer specifications. A microplate pre‐coated with a monoclonal antibody specific for VEGF was provided by the manufacturer. After adding 50 µL of assay diluent, 200 µL of cell culture supernatant were applied to the ELISA plate and incubated for 2 hours at room temperature. Afterwards, a wash step was performed, 200 µL of peroxidase‐linked polyclonal antibody specific for VEGF were added and incubated at room temperature for 2 hours. Another washing step followed. Finally, the substrate solution was applied and incubated 20 minutes protected from light. After adding the stop solution, the absorbance was measured using an ELISA Reader at 450 nm.

### 2.10 RNA isolation, reverse transcription and quantitative real‐time PCR

Total RNA was isolated using the RNeasy^®^ Mini Kit (QIAGEN) according to manufacturer protocol. Final RNA concentration was determined by using NanoDrop (Thermo Scientific). cDNA was synthesized from 1 µg RNA by reverse transcription using QuantiTect^®^ Reverse Transcription Kit (QIAGEN), according to manufacturer specifications. qRT‐PCR was performed using the SsoAvanced™ Universal SYBR^®^ Green Supermix (Bio‐Rad Laboratories), 25 ng cDNA and RPL13a as a reference gene. All primers were designed with the NCBI gene database and purchased from Sigma (Table 1). Data were analyzed by using the relative standard curve method.^27^

### 2.11 Statistical analysis

Graph Pad Prism 7 (Graph Pad Software) was used for statistical analysis. Firstly, the data were tested for normal distribution, using Shapiro‐Wilk test. Afterwards, a Tukey or Kruskal‐Wallis test was performed for multiple comparisons. Data were shown as mean arbitrary units ± standard deviation. *P* values ≤ 0.05 were defined as statistically significant.

## 3 RESULTS

### 3.1 Proliferation

**FIGURE 2:**
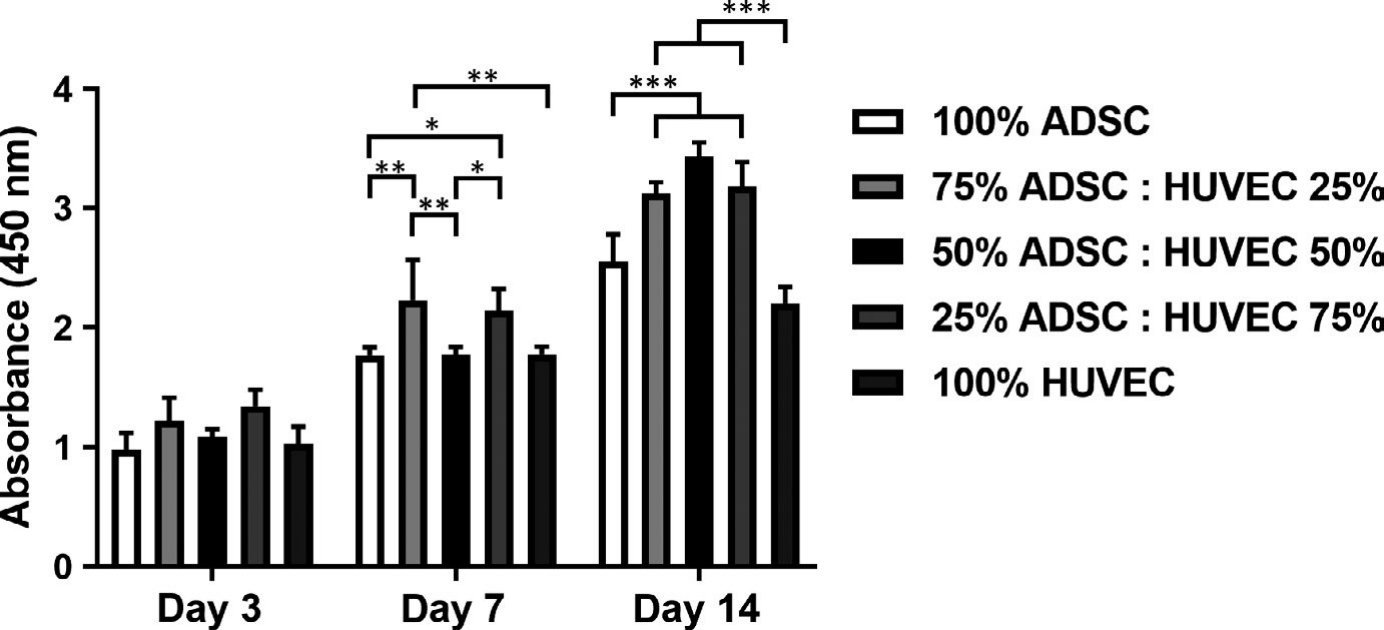
Coculturing ADSCs and HUVECs increased proliferation after 7 and 14 days compared to the monocultures. Statistically significant differences between the experimental groups are indicated for *P ≤ 0.05, **P ≤ 0.01 and ***P ≤ 0.001

A WST‐8 assay was performed as a surrogate assay for cell proliferation upon 3, 7 and 14 days. Over 14 days, the number of vital cells increased in all groups (Figure 2). After 7 days, the amount of vital cells increased in the coculture groups containing 75 or 25% ADSCs. After 14 days, we were able to prove statistically significant more vital cells in all coculture groups compared to the monocultures.

### 3.2 Apoptosis

**FIGURE 3:**
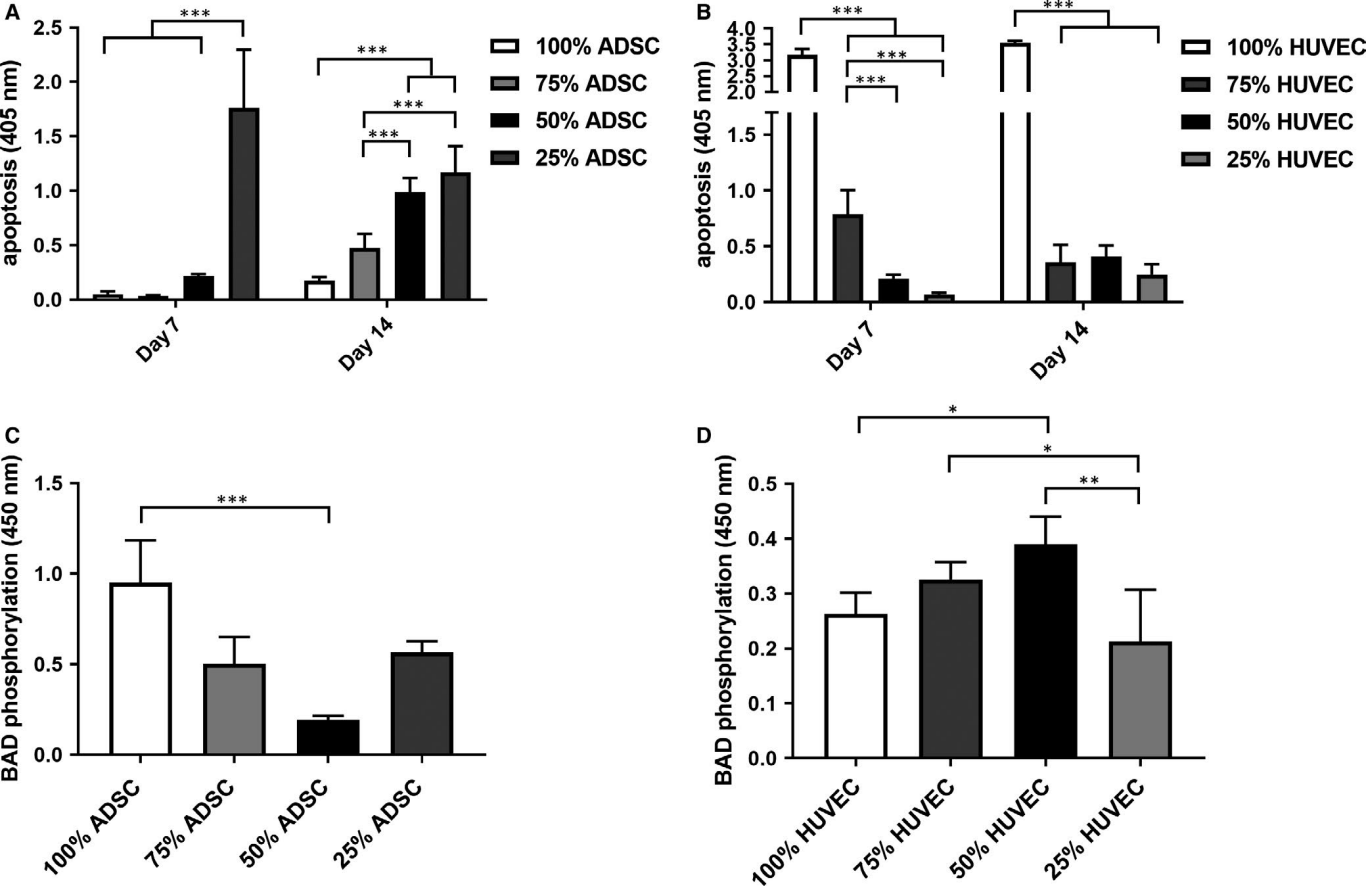
Coculturing ADSCs and HUVECs reduced apoptosis in the HUVECs, whereas apoptosis increased in ADSCs (A, B). The amount of phosphorylated protein BAD was assessed in ADSCs and HUVECs upon coculture demonstrating higher phosphorylated BAD amounts in cocultured HUVECs (C, D). Statistically significant differences are indicated for *P ≤ 0.05, **P ≤ 0.01 and ***P ≤ 0.001

Proportional to the HUVEC ratio, we measured an increasing apoptosis rate in ADSCs with the highest values in the coculture group with 75% ADSCs after 7 and 14 days (Figure 3A). Conversely, HUVECs displayed an unidirectional reduction of apoptosis upon coculture with ADSCs. Interestingly, apoptosis was even more reduced in cocultures with an ADSC ratio ≥50% in the first week. After 2 weeks, we were not able to detect any significant influence of the ADSC ratio on apoptosis in HUVECs (Figure 3B). To enlighten a putative mechanism, we performed a phospho‐BAD ELISA confirming a lower proportion of phosphorylated BAD which might explain the higher apoptosis rate in cocultured ADSCs. On the other hand, we found increasing levels of phosphorylated BAD in cocultured HUVECs leading to decreased apoptosis (Figure 3C,D).

### 3.3 Osteogenic differentiation

**FIGURE 4:**
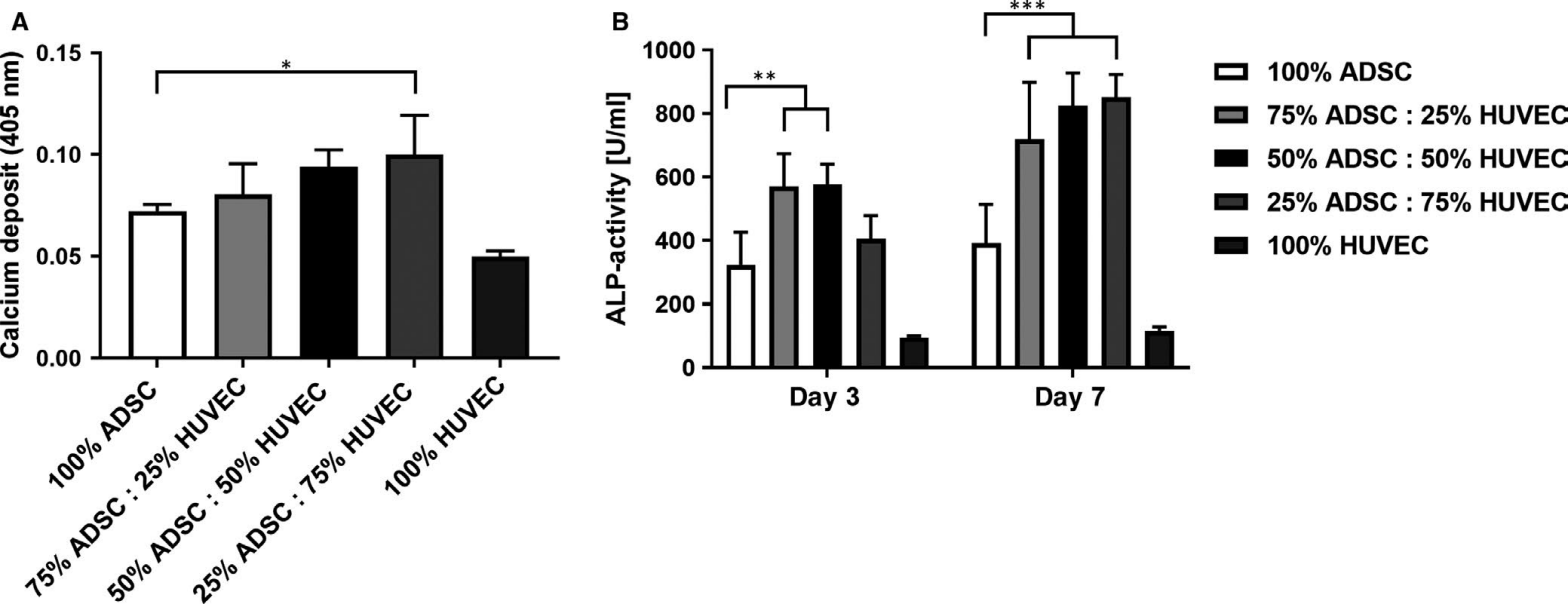
Alizarin red assay measuring matrix mineralization (A). Alkaline phosphatase (ALP) activity was measured as a surrogate parameter for osteogenic differentiation. Higher ALP activity was found in the cocultures upon 3 and 7 days (B). Statistically significant differences are indicated for *P ≤ 0.05, **P ≤ 0.01 and ***P ≤ 0.001

Coculturing ADSCs and HUVECs increased matrix mineralization as proved by alizarin red assay (Figure 4A). Moreover, matrix mineralization increased with higher HUVEC ratios in the coculture groups. Alkaline phosphatase (ALP) activity is another surrogate parameter for osteogenic differentiation. On day 3, we observed an induction of ALP activity in the coculture groups with ≥25% HUVECs. After 7 days, this effect was even more pronounced in all cocultures (twofold induction) (Figure 4B).

**FIGURE 5:**
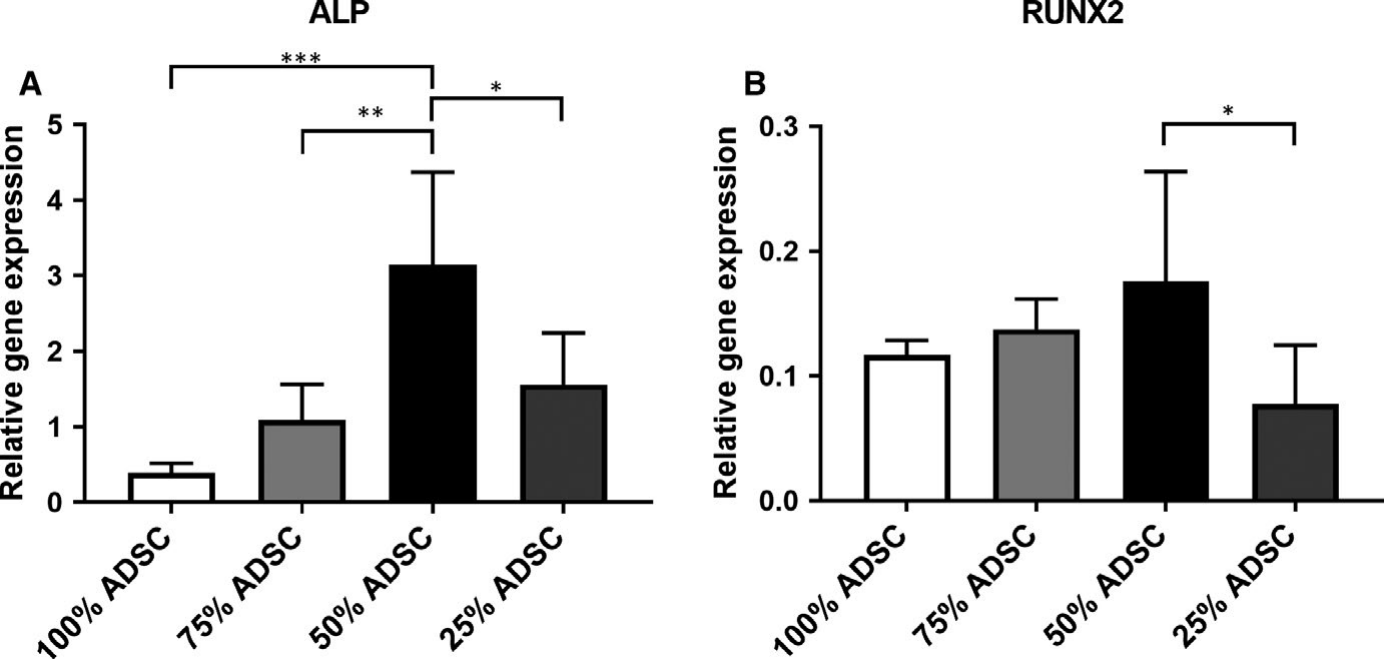
PCR analysis concerning ALP and RUNX2 gene expression after negative immunoseparation. In the coculture group containing 50% ADSCs, the expression of ALP increased significantly (A). A same trend towards higher RUNX2 expression was also observed in cocultured ADSCs (B). Statistically significant differences are indicated for *P ≤ 0.05, **P ≤ 0.01 and ***P ≤ 0.001

After negative immunoselection and PCR, we were able to prove that the induction of ALP gene expression is limited to ADSCs. Additionally, we measured the highest ALP mRNA levels in the coculture group with 50% ADSCs (Figure 5A). Moreover, we found increasing levels of the osteogenic transcription factor RUNX2 in ADSCs after coculturing. The coculture with 50% ADSCs displayed the highest RUNX2 upregulation (Figure 5B).

### 3.4 Angiogenesis

**FIGURE 6:**
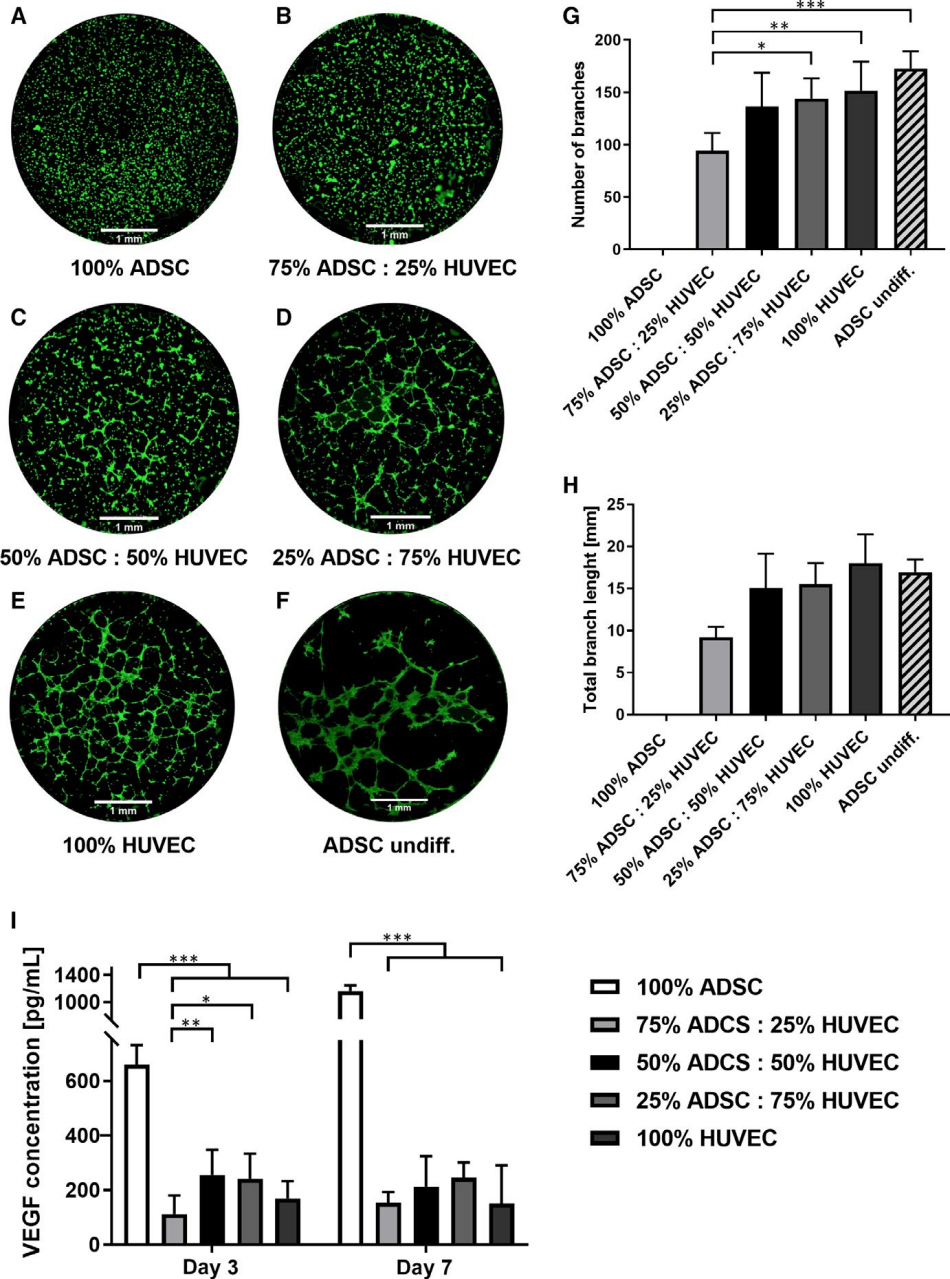
Matrigel assay demonstrating that the number of tubes (G) as well as the total tube length (H) increased with the HUVEC ratio. In addition to that, undifferentiated ADSCs (F) formed tubes. VEGF ELISA depicts the highest VEGF production in ADSCs under monoculture conditions (I). Statistically significant differences are indicated for *P ≤ 0.05, **P ≤ 0.01 and ***P ≤ 0.001

**FIGURE 7:**
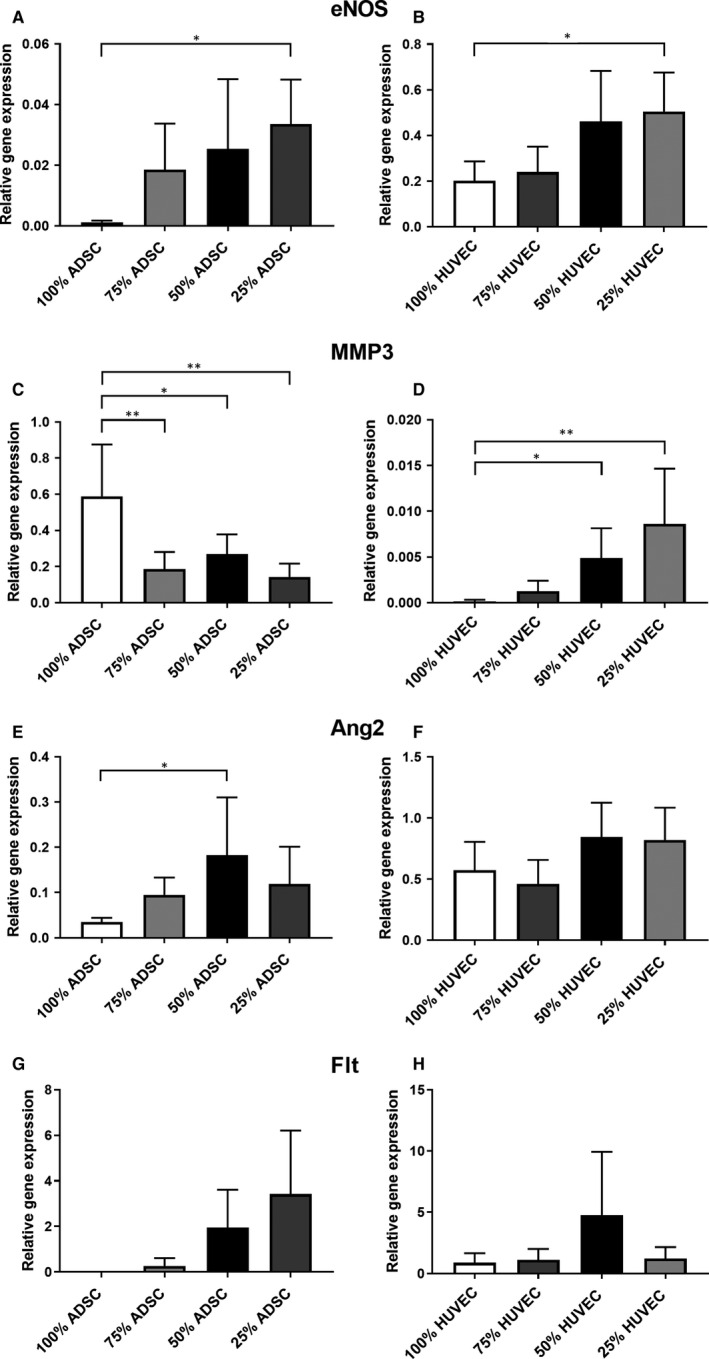
PCR analysis of cocultured ADSCs and HUVECs after negative immunoseparation. The gene expression of eNOS (A), Ang2 (E) and Flt (G) is higher in cocultured ADSCs. eNOS (B) and MMP3 (D) are higher expressed in cocultured HUVECs, whereas a lower MMP3 (C) gene expression was observed in cocultured ADSCs. Statistically significant differences are indicated for *P ≤ 0.05 and **P ≤ 0.01

To investigate the angiogenic potential of ADSC/HUVEC cocultures, matrigel assays were performed. On the contrary to osteogenic differentiated ADSCs, undifferentiated ADSCs formed tubes in matrigel. Proportionally to the amount of HUVECs, the number and length of tubes increased in the cocultures (Figure 6A‐H). Using VEGF ELISA, we were able to prove the highest VEGF production in ADSCs under monoculture conditions on days 3 and 7. Moreover, the production of VEGF was even more pronounced in cocultures containing ≥50% HUVECs on day 3. No statistically significant differences were detected between the cocultures on day 7 (Figure 6I). Gene expression of angiogenic molecules, such as endothelial nitric oxidase (eNOS) or matrix metalloprotease 3 (MMP3) increased in the cocultured HUVECs proportionally to higher ADSC ratios (Figure 7B,D).

Furthermore, cocultivation stimulated the gene expression of endothelial nitric oxidase (eNOS), angiopoetin 2 (Ang2) and VEGF receptor 1 (Flt) in ADSCs (Figure 7A,E,G), whereas MMP3 gene expression was downregulated (Figure 7C).

## 4 DISCUSSION

Osteogenesis and angiogenesis are two directly related processes. A stable microvascular network, providing an adequate supply of oxygen and nutrients, is essential for bone formation and regeneration.^9^, ^28^ For this reason, the interaction between mesenchymal stem cells and endothelial cells has been widely studied demonstrating auspicious effects concerning cell growth, survival and osteogenic differentiation.^10^, ^11^, ^29^, ^30^ Although the molecular mechanisms are not fully understood, heterotypic cell contacts between mesenchymal stem cells and endothelial cells seem to play an important role.^10^, ^11^, ^30^, ^31^, ^32^, ^33^ So far, most studies used MSCs or osteoprogenitor cells isolated from bone marrow. Considerably fewer studies used MSCs isolated from fat tissue. However, ADSCs can be isolated in sufficient quantity without significant donor side morbidity and expanded in vitro.^34^, ^35^, ^36^ In several in vivo studies, ADSCs were used to reconstruct critical sized bone defects.^37^, ^38^, ^39^, ^40^

Considering the fact that most studies used rodent cells and/or focused on global effects of coculturing ADSCs and endothelial cells, we pursued a translational approach using human primary cells and described cell type specific effects upon coculturing using negative immunoselection. In addition to that, we tried to determine the ideal cell ratio of ADSCs and HUVECs with regard to proliferation, apoptosis, osteogenic differentiation and angiogenesis.

According to previous studies, our results indicate that cell proliferation increased in all groups over 14 days. Moreover, the proliferation rate was significantly higher in the cocultures after 7 and 14 days, especially if the cell ratio constituted >50% ADSCs.^30^, ^33^ We also investigated apoptosis in the cocultures after 7 and 14 days. Using negative immunoseparation, we were able to analyze apoptosis of ADSCs and HUVECs separately. Our results indicate an anti‐apoptotic effect of ADSCs on HUVECs. This effect was even more pronounced with an increasing ADSC ratio. Contrary to a previous study using HUVECs and MSCs, HUVECs did not reduce apoptosis in ADSCs.^30^ In fact, apoptosis increased in cocultured ADSCs. A possible explanation for this unexpected phenomenon would be that ADSCs are more vulnerable than HUVECs to increasing cell density. In this respect, Kim et al have already described that proliferation and stem cell properties of ADSCs are strongly dependent on cell density.^41^ Another explanation for increased apoptosis in cocultured ADSCs might be the lower VEGF amount in the supernatant compared to ADSCs under monoculture conditions.^42^ The proapoptotic protein BAD plays an important role in regulation of cell death. Phosphorylation of BAD at Ser112 and/or Ser136 inhibits programmed cell death.^43^, ^44^ Coincident with higher phospho‐BAD levels, we were able to prove a lower apoptosis rate in cocultured HUVECs. In this regard, HUVECs cocultured with 50% ADSCs displayed the highest phospho‐BAD level. In accordance with higher apoptosis rates in cocultured ADSCs, we found a lower phosphorylation of BAD. It is alluring to speculate that phosphorylation of BAD mediates apoptosis in cocultured HUVECs.

Previous studies demonstrated a stimulation of osteogenic differentiation of MSCs or osteogenic progenitor cells upon cocultivation with endothelial cells.^9^, ^11^ In our study, we were able to prove higher matrix mineralization in the coculture groups after 14 days. This effect was even more pronounced with higher HUVEC amounts (≥50%). The alkaline phosphatase (ALP) is an early marker of osteogenic differentiation. Our results demonstrated a significantly increased ALP activity in the cocultures. Using negative immunoseparation, we were able to prove that HUVECs stimulated gene expression of ALP in ADSCs with the highest gene expression in the coculture group containing 50% ADSCs. The transcription factor RUNX2 is also known as a hall mark of osteogenesis because RUNX2 promotes gene expression of other osteogenic differentiation markers such as ALP and OCN.^45^ In consistence with increasing HUVEC cell ratios, we observed a stronger effect on RUNX2 gene expression in cocultured ADSCs. Although not statistically significant, the highest RUNX2 gene expression was found in the coculture with 50% HUVECs. In summary, HUVECs promote osteogenic differentiation of ADSCs and a ratio of 50% HUVECs seems to be advantageous.

Bearing in mind that angiogenesis and osteogenesis are two directly linked processes in terms of bone regeneration, we wanted to enlighten the angiogenic potential of ADSC/HUVEC cocultures. In the pertinent literature, the angiogenic potential of ADSCs on endothelial cells is controversially discussed.^46^, ^47^, ^48^ In our experiments, we were able to prove proangiogenic effects upon coculturing ADSCs and HUVECs correlating with the amount of HUVECs. In this regard, no sprouting was observed in the osteogenic differentiated ADSC monoculture. Interestingly, we observed sprouting in the undifferentiated ADSC monoculture as well in cocultures containing HUVECs.

Polymerase Chain Reaction analysis displayed a significant upregulation of the proangiogenic signal molecule endothelial nitric oxide synthase (eNOs) in HUVECs and ADSCs. It is well known that circulating signal molecules activate protein kinase B and eNOS thereby promoting angiogenesis.^49^ More precisely, by releasing the vasodilator NO the eNOS regulates the vascular tone but also enhances the formation of new vessels.^50^, ^51^ Vascular endothelial cell growth factor (VEGF) is another important proangiogenic signal molecule. Our results indicate the highest VEGF levels in ADSCs under monoculture conditions on days 3 and 7. However, a closer look at day 3 reveals that VEGF production increases with higher HUVEC ratios (≥50%) in the cocultures. Interestingly, we found no VEGF‐R1 (Flt) gene expression in the ADSC monoculture but enhanced Flt gene expression in the cocultures containing ≥50% HUVECs. As discussed by Chen et al, VEGF is a potent mitogen and it is alluring to speculate that ADSCs respond to lower VEGF levels with increased Flt gene expression.^52^ Another promising growth factor is angiopoetin 2 (Ang2), which is significantly upregulated in cocultured ADSCs. Ang 2 plays a critical role in angiogenesis and supports bone healing in rabbits.^53^ Angiogenesis is a well‐orchestrated process supported by extracellular matrix degradation and migration mediated by matrix metalloproteases.^54^, ^55^ In our experiments, MMP‐3 gene expression in HUVECs correlated with increasing ADSC ratios of ≥50% but not vice versa. Consistent with the results from the matrigel assay, one gets the impression that a minimum of 50% HUVECs is necessary to stimulate proangiogenic effects of cocultured ADSCs.

In conclusion our results have shown that proliferation, osteogenic differentiation and proangiogenic features are significantly enhanced in cocultures of ADSCs and HUVECs, especially if a cell ratio of 50% ADSCs: 50% HUVECs was used. Furthermore, apoptosis was decreased unidirectional in HUVECs, mediated by a phospho‐BAD‐dependent mechanism. Future in vivo studies are necessary to investigate these promising in vitro effects in terms of bone tissue engineering. For this purpose, osteogenic scaffolds containing ADSCs and HUVECs will be implanted in the rat AV loop model. The microsurgically induced angiogenesis by means of the AV loop will support adequate vascularization and thereby cell survival providing an excellent translational model for a possible clinical application.^4^, ^56^, ^57^

## CONFLICT OF INTEREST

The authors confirm that there are no conflicts of interest.

## AUTHOR CONTRIBUTIONS

DS, REH and AA made substantial contributions to conception and design of the study. HM, DS, SW and VW made substantial contributions to acquisition, analysis and interpretation of data. DS, HM, SW, REH, AA and VW were involved in drafting the manuscript or revising it critically for important intellectual content. All authors have given final approval of the manuscript.

## DISCLOSURE

The present work was performed in fulfilment of the requirements for obtaining the degree “Dr. med.” of Hilkea Mutschall.

## Data Availability

The data that support the findings of this study are available from the corresponding author upon reasonable request.
